# Genome wide association study identifies SNPs associated with fatty acid composition in Chinese Wagyu cattle

**DOI:** 10.1186/s40104-019-0322-0

**Published:** 2019-03-04

**Authors:** Zezhao Wang, Bo Zhu, Hong Niu, Wengang Zhang, Ling Xu, Lei Xu, Yan Chen, Lupei Zhang, Xue Gao, Huijiang Gao, Shengli Zhang, Lingyang Xu, Junya Li

**Affiliations:** 1 Innovation Team of Cattle Genetic Breeding, Institute of Animal Sciences, Chinese Academy of Agricultural Sciences, Beijing, 100193 China; 2 National Engineering Laboratory for Animal Breeding, Key Laboratory of Animal Genetics, Breeding and Reproduction, Ministry of Agriculture, College of Animal Science and Technology, China Agricultural University, Beijing, 100193 China; 3 Institute of Animal Husbandry and Veterinary Research, Anhui Academy of Agricultural Sciences, Hefei, 230031 China

**Keywords:** Chinese Wagyu cattle, Fatty acids, GWAS, Meat quality, Pleiotropic effects

## Abstract

**Background:**

Fatty acids are important traits that affect meat quality and nutritive values in beef cattle. Detection of genetic variants for fatty acid composition can help to elucidate the genetic mechanism underpinning these traits and promote the improvement of fatty acid profiles. In this study, we performed a genome-wide association study (GWAS) on fatty acid composition using high-density single nucleotide polymorphism (SNP) arrays in Chinese Wagyu cattle.

**Results:**

In total, we detected 15 and 8 significant genome-wide SNPs for individual fatty acids and fatty acid groups in Chinese Wagyu cattle, respectively. Also, we identified nine candidate genes based on 100 kb regions around associated SNPs. Four SNPs significantly associated with C14:1 *cis*-9 were embedded with stearoyl-CoA desaturase (*SCD*), while three SNPs in total were identified for C22:6 n-3 within Phospholipid scramblase family member 5 (*PLSCR5*), Cytoplasmic linker associated protein 1 (*CLASP1*), and Chymosin (*CYM*). Notably, we found the top candidate SNP within *SCD* can explain ~ 7.37% of phenotypic variance for C14:1 *cis*-9. Moreover, we detected several blocks with high LD in the 100 kb region around *SCD*. In addition, we found three significant SNPs within a 100 kb region showing pleiotropic effects related to multiple FA groups (PUFA, n-6, and PUFA/SFA), which contains BAI1 associated protein 2 like 2 (*BAIAP2L2*), MAF bZIP transcription factor F (*MAFF*), and transmembrane protein 184B (*TMEM184B*).

**Conclusions:**

Our study identified several significant SNPs and candidate genes for individual fatty acids and fatty acid groups in Chinese Wagyu cattle, and these findings will further assist the design of breeding programs for meat quality in cattle.

**Electronic supplementary material:**

The online version of this article (10.1186/s40104-019-0322-0) contains supplementary material, which is available to authorized users.

## Background

Fatty acids of beef products have received considerable attention for their significance in human health, the improvement of salutary fatty acid (FA) content can offer more economic benefits in the beef market [1–6]. Like most economically important traits in beef cattle, FA composition are complex traits influenced by both genetic and environmental factors. Several studies have suggested that FA composition are lowly or moderately heritable traits and can be altered by feeding strategies [7–10]. However, recent studies from estimates of genetic parameters suggested that investigation of the genetic basis for FA composition can enable us to promote genetic improvement for them [7, 9, 11, 12]. Several studies have also reported genetic parameter analyses and genome-wide association studies of fatty acid profiles from milk in dairy cattle [13, 14]. Application of molecular genetic approaches can provide more opportunities to design genomic selection strategies for meat quality in beef cattle [15, 16].

In the last decade, genome-wide association studies (GWASs) have emerged as a powerful approach for detecting the candidate variants and genes for complex traits in beef cattle [8, 17–19]. Many studies have identified candidate markers associated with FA composition in different populations, such as Japanese Black cattle, Hereford, Angus, and Shorthorn [16, 20–23]. However, most of these studies were carried out using low density SNP arrays. Only a few studies were conducted using the high-density BovineHD (770 K) SNP array in limited breeds [8, 24, 25]. The genetic basis of FA composition may vary among populations, and the use of genomic technologies for improving fatty acid profiles in beef cattle have not been comprehensively addressed [8, 16, 21, 23–29].

Wagyu is especially well-known for its remarkable marbling score and meat quality [6, 30]. While the marbling is mainly fat tissue containing abundant monounsaturated fatty acids (MUFAs), it also reflects a lower melting point and contributes positively to beef flavor and tenderness [6, 31]. Several studies have been reported to investigate the genetic basis of fatty acids in Wagyu cattle, and a list of associated SNPs and candidate genes have been identified for these traits [6, 16, 32–34]. Chinese Wagyu cattle is a hybrid population from Wagyu and Fuzhou cattle, Fuzhou cattle is an indigenous population raised in Liaoning Province, China. Therefore, a GWAS of Chinese Wagyu cattle can contribute valuable knowledge for understanding the genetic basis of fatty acid composition. The objectives of the current study were to 1) identify the associated genomic variants and relative candidate genes for FA composition and 2) elucidate the genetic architecture of FA composition at the whole genome level in Chinese Wagyu cattle.

## Materials and methods

### Ethics statement

All animals were treated following the guidelines established by the Council of China Animal Welfare. Experimental protocols were approved by the Science Research Department of the Institute of Animal Sciences, Chinese Academy of Agricultural Sciences (CAAS) (Beijing, China).

### Animals and phenotypes

The Chinese Wagyu population (464 animals) was established in Dalian, Liaoning Province, China, and all animals were born between 2012 and 2013. After weaning, they were fattened using the same feeding conditions for 20–24 months and slaughtered at an average of 28 months. During slaughtering, we measured meat quality traits in strict accordance with the guidelines proposed by the Institutional Meat Purchase Specifications for fresh beef. Meat samples were selected from the longissimus lumborum muscle, between the 12^th^ and 13^th^ ribs from each animal, after storage for 48 h, and samples were vacuum packed and chilled at − 80 °C. In addition, approximately 10 g of sample were taken for subsequent analyses. Total lipids were extracted from samples according to protocols described previously [35]. Approximately 2 mg of extracted lipid was re-dissolved in 2 mL of n-hexane and 1 mL of KOH (0.4 mol/L) for saponification and methylation. A total of 21 individual fatty acids, including six saturated fatty acids, four monounsaturated FAs, and eleven polyunsaturated fatty acids, were measured using gas chromatography (GC-2014 CAFsc, Shimadzu Scientific Instruments). Each FA was quantified as a weight of percentage of total FAs. In addition, FAs groups were measured as total saturated fatty acid (SFA), total monounsaturated (MUFA), total polyunsaturated (PUFA), total omega 3 (n-3), total omega 6 (n-6), ratio between PUFA and SFA (PUFA/SFA), ratio between n-6 and n-3 (n-6/n-3) and health index (HI). The estimation of various FA groups follows the same process as previously described [8, 11].

### Genotyping and quality control

Blood samples were obtained with the regular quarantine inspection of the farms. DNA was extracted from the blood samples using a routine procedure. In total, 464 individuals were genotyped using the Illumina Bovine HD BeadChip (Illumina, Inc., San Diego, CA). SNPs were pre-processed based on the following filters using PLINK v1.07 [36]: Minor allele frequency (> 0.05), proportion of missing genotypes (< 0.05), and the Hardy-Weinberg equilibrium (*P* > 10E-6). Moreover, individuals with more than 10% missing genotypes were excluded. After quality control, the final data consisted of 364 individuals and 503,579 autosomal SNPs.

### Heritability and genetic correlation estimation

Phenotypic and genetic (co)variances of fatty acids were estimated using the pairwise bivariate animal model implemented in the ASReml v3.0 package [37]. The model is

$$ \left[\begin{array}{c}{y}_1\\ {}{y}_2\end{array}\right]=\left[\begin{array}{cc}{X}_1& 0\\ {}0& {X}_2\end{array}\right]\left[\begin{array}{c}{b}_1\\ {}{b}_2\end{array}\right]+\left[\begin{array}{cc}{Z}_1& 0\\ {}0& {Z}_2\end{array}\right]\left[\begin{array}{c}{a}_1\\ {}{a}_2\end{array}\right]+\left[\begin{array}{c}{e}_1\\ {}{e}_2\end{array}\right] $$

where *y*_1_ and *y*_2_ are vectors of phenotypic values of trait 1 and 2, respectively; *X*_1_ and *X*_2_ are incidence matrices for fixed effects; *b*_1_ and *b*_2_ are vectors of the fixed effects; *Z*_1_ and *Z*_2_ are incidence matrices relating the phenotypic observations to vectors of the polygenic (*a*_*1*_ *+ a*_*2*_) effects for two traits; and *e*_1_ and *e*_2_ are random residuals for two traits. Variances of the random effects are defined as $V(a)=\mathbf{G}{\sigma}_a^2$ for the polygenes and $V(e)=\mathbf{I}{\sigma}_e^2$ for the residuals, where **G** is the additive genetic relationship matrix, **I** is the identity matrix, ${\sigma}_a^2$ is the additive genetic variance, and ${\sigma}_e^2$ is the residual variance. The **G** matrix was constructed using the SNP genotypes based on the proportion of total loci shared by two individuals [38], which was defined as **G** = **MM**^′^/ ∑ 2*p*_*i*_(1 − *p*_*i*_), where **M** is an *n* × *m* matrix of the number of animals (*n*) and number of marker loci (*m*), and it specifies the marker genotype coefficient at each locus, *p*_*i*_ is the frequency of allele A of SNP, and (1 − *p*_*i*_) is the frequency of allele B. In **MM**^′^ the number of alleles shared by relatives was reported on the off-diagonals and an individual’s relationship with itself was reported on the diagonals. Farm and carcass grade were considered as fixed effects in the model. In addition, the duration of fattening and back-fat thickness were considered as covariates in the model. Genomic heritability of each trait was estimated using ${h}^2={\sigma}_a^2/\left({\sigma}_a^2+{\sigma}_e^2\right)$. The phenotypic and genetic correlation coefficients were calculated using ${r}_P={\mathit{\operatorname{cov}}}_{P_{XY}}/\sqrt{\sigma_{P_X}^2\times {\sigma}_{P_Y}^2}$ and${r}_G={\mathit{\operatorname{cov}}}_{G_{XY}}/\sqrt{\sigma_{G_X}^2\times {\sigma}_{G_Y}^2}$, where *r*_*P*_ and *r*_*G*_ are phenotypic and genetic correlation coefficients, respectively. ${\sigma}_{P_X}^2$ and ${\sigma}_{P_Y}^2$ are the phenotypic variance of trait X and trait Y. ${\sigma}_{P_X}^2$ and ${\sigma}_{P_Y}^2$ are the additive genetic variance of trait X and trait Y. ${\mathit{\operatorname{cov}}}_{P_{XY}}$ and ${\mathit{\operatorname{cov}}}_{G_{XY}}$ are the phenotypic and genetic covariance.

### Genome-wide association analysis using FarmCPU

Fatty acid composition were adjusted for fixed effects and covariates using linear mixed models. The Fixed and random model Circulating Probability Unification (FarmCPU) model was used to test the single-SNP association. This algorithm takes into account the confounding problem between covariates using both the Fixed Effect Model (FEM) and Random Effect Model (REM) [39, 40]. The first three principal components were calculated using GAPIT, which were considered as the covariates [41]. The quantile-quantile (Q-Q) plot was generated to assess population stratification [42]. The linkage disequilibrium (*r*^2^) was estimated using PLINK v1.07 software. Linkage disequilibrium between SNPs around the target regions were estimated and visualized using Haploview v4.3 software [43]. Region plots were generated using the *asplot* function in the R package “gap” [44]. Positional candidate genes were investigated for 100 kb windows around SNPs using UCSC Genome Browser, which was based on the *Bos taurus* genome assembly UMD 3.1. The proportion of phenotypic variance explained by each SNP was calculated as follows:

$$ {V}_{pi}=\frac{2{p}_i{q}_i{\upbeta}_i^2}{\sigma_p^2}\times 100\% $$

where *p*_*i*_ and *q*_*i*_ represent the frequencies of two alleles for the *i*^th^ SNP, *β*_*i*_ is the effect of the *i*^th^ SNP, and ${\sigma}_p^2$ denotes the phenotypic variance.

## Results

### Descriptive statistics and heritability estimations of fatty acid compositions

Descriptive statistics and heritability estimate results were presented in Table 1 for 21 individual FAs and eight FA groups. We observed that the most abundant individual saturated FAs were C18:1 *cis*-9 (46.87%), and C16:0 (27.46%), while relatively high proportions of the total FAs were found for C18:0 (11.96%), C16:1 *cis*-9 (4.22%), and C18:2 n-6 (3.84%) among the 21 individual FAs. In contrast, saturated FAs (C20:0, C22:0, C24:0), monounsaturated FAs (C14:1 *cis*-9, C20:1 *cis*-11), and polyunsaturated FAs (C18:2 c-9 t-11, C18:2 c-12 t-10, C18:3 n-6, C18:3 n-3, C20:2 n-6, C20:3 n-3, C20:4 n-6, C20:5 n-3, C22:5 n-3, C22:6 n-3) occupy relatively low proportions. For fatty acid groups, Saturated fatty acids (SFA), monounsaturated fatty acids (MUFA), and polyunsaturated fatty acids (PUFA) account for 42.5%, 52.51%, and 4.96% of total FAs, respectively. In general, the relative proportions of FAs and FA groups were consistent with our previous findings in Chinese Simmental cattle [8].

**Table 1:** Summary statistics of mean (%), standard deviation (SD, %) and heritability estimates (*h*^2^), additive genetic variance and coefficient of variation (CV, %)

Trait ^a^	Name	Mean ± SD	Additive genetic variance	Residual variance	CV, %	*h*^2^ ± SE
Saturated FA						
C14:0	Myristic	2.97 ± 0.6	1.24E-01	2.42E-01	20.31	0.34 ± 0.16
C16:0	Palmitic	27.46 ± 4.06	8.35E-02	1.64E+ 01	14.78	0.01 ± 0.07
C18:0	Stearic	11.96 ± 2.65	1.32E+ 00	4.88E+ 00	22.12	0.21 ± 0.13
C20:0	Arachidic	0.08 ± 0.02	8.60E-06	4.01E-04	24.74	0.02 ± 0.07
C22:0	Behenic	0.02 ± 0.01	9.10E-06	2.63E-05	36.16	0.26 ± 0.16
C24:0	Methyl tetracosanoate	0.04 ± 0.02	3.37E-05	4.44E-04	58.88	0.07 ± 0.12
Monounsaturated FA						
C14:1 *cis*-9	Methyl myristoleate	0.93 ± 0.3	3.77E-02	5.02E-02	32.23	0.43 ± 0.17
C16:1 *cis*-9	Methyl palmitoleate	4.22 ± 0.94	5.97E-02	7.63E-01	22.17	0.07 ± 0.12
C18:1 *cis*-9	Oleic	46.87 ± 4.48	5.12E+ 00	1.53E+ 01	9.55	0.25 ± 0.17
C20:1 *cis*-11	*cis*-11-eicosenoic acid methyl ester	0.48 ± 0.14	3.10E-03	1.53E-02	28.99	0.17 ± 0.11
Polyunsaturated FA						
C18:2 n-6	Linoleic	3.84 ± 0.74	1.10E-02	5.01E-01	19.36	0.02 ± 0.07
C18:2 c-9 t-11	*cis*-9 and *trans*-11 octadecadienoic acid methyl esters	0.3 ± 0.09	2.00E-03	1.14E-01	30.23	0.02 ± 0.19
C18:2 c-12 t-10	*cis*-12 and *trans*-10 octadecadienoic acid methyl esters	0.03 ± 0.01	3.14E-05	1.30E-04	54.44	0.19 ± 0.14
C18:3 n-6	Methyl γ-linoleate	0.08 ± 0.02	8.96E-05	3.00E-03	24.62	0.03 ± 0.33
C18:3 n-3	Linolenic	0.24 ± 0.06	3.00E-04	2.90E-03	24.67	0.09 ± 0.11
C20:2 n-6	*cis*-11,14-eicosadienoic acid methyl ester	0.06 ± 0.02	2.85E-05	2.53E-04	30.08	0.1 ± 0.09
C20:3 n-3	*cis*-11,14,17-eicosatrienoic acid methyl ester	0.26 ± 0.14	1.90E-03	8.10E-03	53.31	0.19 ± 0.14
C20:4 n-6	*cis*-5,8,11,14-eicosatetraenoic acid methyl ester	0.01 ± 0	3.70E-06	1.24E-05	49.12	0.23 ± 0.15
C20:5 n-3	*cis*-5,8,11,14,17-eicosapentaenoic acid methyl ester	0.07 ± 0.03	4.09E-05	7.32E-04	41.3	0.05 ± 0.08
C22:5 n-3	*cis*-7,10,13,16,19-docosapentaenoic methyl ester	0.06 ± 0.03	2.28E-04	1.50E-02	51.35	0.02 ± 0.38
C22:6 n-3	*cis*-4,7,10,13,16,19-docosahexaenoic acid methyl ester	0.02 ± 0.02	1.00E-04	2.00E-04	69.71	0.3 ± 0.17
Fatty acid groups						
SFA	Total saturated fatty acid	42.53 ± 3.61	6.91E+ 00	6.68E+ 00	8.48	0.51 ± 0.16
MUFA	Total monounsaturated fatty acid	52.51 ± 4.05	6.73E+ 00	9.91E+ 00	7.72	0.4 ± 0.17
PUFA	Total polyunsaturated fatty acid	4.96 ± 0.92	1.04E-01	6.68E-01	18.51	0.13 ± 0.15
PUFA/SFA	Ratio between PUFA and SFA	0.12 ± 0.02	7.45E-05	2.72E-04	16.84	0.21 ± 0.14
n-3	Total omega-3 fatty acids	0.65 ± 0.18	1.40E-03	1.92E-02	27.49	0.07 ± 0.1
n-6	Total omega-6 fatty acids	3.99 ± 0.76	7.70E-02	4.79E-01	19.12	0.14 ± 0.13
n-6/n-3	Ratio between n-6 and n-3	6.37 ± 1.17	3.19E-01	6.75E-01	18.39	0.32 ± 0.16
HI	Health index	1.49 ± 0.24	2.50E-02	3.31E-02	15.73	0.43 ± 0.17

^a^The concentrations of fatty acids were expressed as a percentage of total fatty acid methyl esters quantified

The heritability varied remarkably among 21 individual fatty acids. Among them, C14:1 *cis*-9 showed the highest heritability (0.43), while six fatty acids, including C14:0, C18:0, C22:0, C18:1 *cis*-9, C20:4 n-6, and C22:6 n-3, displayed moderate heritability (0.2~0.4). The estimated heritability for 14 out of 21 other FAs were less than 0.2. For eight FAs groups, high heritability were estimated for SFA, health index (HI), and MUFA (0.51, 0.43 and 0.40), while the estimated heritability for PUFA, total omega 3, and total omega 6 were 0.13, 0.07, and 0.14, respectively.

### Phenotypic and genetic correlations between fatty acids

We estimated the phenotypic and genetic correlations among 21 individual FAs, which were shown in Additional file 1: Table S1. For six individual SFAs, high positive phenotypic and genetic correlations were observed between each pairwise comparison of C14:0, C16:0, and C18:0. Strong positive genetic correlations for C20:0 vs. C22:0 (0.994) and C20:0 vs. C24:0 (0.990) were observed, while strong negative genetic correlations between C22:0 and C24:0 was −0.99. Also, we observed a weak correlation between C20:0 vs. C14:0 (−0.098) and C20:0 vs. C16:0 (0.013) (Table S1). For individual MUFA, methyl myristoleate (C14:1 *cis*-9) showed a high positive genetic correlation with C16:1 *cis*-9 (0.866), C18:1 *cis*-9 (0.789), and C20:1 *cis*-11 (0.864). Also, C18:1 *cis*-9 has a highly positive genetic correlation with C16:1 *cis*-9 (0.839). For individual PUFA, several highly positive and negative genetic correlations were estimated among these individual groups. For instance, high positive correlations were observed among C22:6 n-3 vs. C22:5 n-3 (0.992), C22:6 n-3 vs. C24:0 n-6 (0.99), C18:3 n-3 vs. C20:3 n-3 (0.99), and C20:2 n-6 vs. C14:1 *cis*-9 (0.991). In contrast, negative correlations were observed among C18:3 n-3 vs. C20:5 n-3 (−0.976), C20:2 n-6 vs. C20:5 n-3 (−0.966), and C20:4 n-6 vs. C20:5 n-3 (−0.978).

### GWAS results and candidate regions

We performed GWAS for 21 individual FAs and 8 FA groups using FarmCPU, and only results for traits with genomic heritability ≥0.10 were reported in the current study. As the Bonferroni correction was highly conservative for the GWAS using the high-density SNP array, we considered *P* < 1.36E-06 (0.1/73,531) as the suggestive significant level which was proposed by Zhu et al. [8] and Duggal et al. [45]. This strategy evaluates the approximate number of “independent” SNPs by counting one SNP per linkage disequilibrium (LD) block, plus all SNPs outside of the LD blocks (inter-block SNPs). The summary of the results from the GWAS using FarmCPU methods were shown in Table 2. In total, we identified 15 and 8 candidate SNPs for individual FAs and FA groups, respectively.

**Table 2:** Candidate regions associated with fatty acid composition and fatty acid groups

Trait	SNP name	BTA	Position	SNP^a^	MAF	*P-*value	SNP effect^b^	SE	PCG^c^
Saturated FA									
C22:0	BovineHD0600017622	6	63657334	G/A	0.342	5.80E-07	0.002212982	0.000454901	–
Monounsaturated FA									
C14:1 *cis*-9	BovineHD2600005461	26	21140458	G/A	0.3791	1.72E-07	−0.116976244	0.0247878	*SCD*
C14:1 *cis*-9	BovineHD2600005464	26	21144884	G/A	0.3764	2.62E-07	−0.114923399	0.0247513	*SCD*
C14:1 *cis*-9	BovineHD2600005466	26	21146794	G/A	0.3984	1.02E-07	−0.118178451	0.0242669	*SCD*
C14:1 *cis*-9	BovineHD2600005465	26	21146019	G/A	0.397	1.22E-07	−0.117257801	0.0241962	*SCD*
C18:1 *cis*-9	BovineHD0600017761	6	64216527	A/G	0.0989	2.90E-07	−2.77403258	0.55471	*–*
C18:1 *cis*-9	BovineHD0600017773	6	64280345	C/A	0.1511	4.12E-07	−2.356278809	0.477168	*–*
C20:1 *cis*-11	BovineHD0800012682	8	42650702	G/A	0.05769	4.89E-07	0.105893342	0.00215886	*SMARCA2*
Polyunsaturated FA									
C22:6 n-3	BovineHD0100034705	1	122808627	G/A	0.364	3.38E-10	0.004481756	0.00137556	*PLSCR5*
C22:6 n-3	BovineHD0200040673	2	73693578	G/A	0.0728	9.19E-08	0.007072896	0.00233111	*CLASP1*
C22:6 n-3	BovineHD0300010307	3	33051872	A/G	0.06319	1.31E-07	0.007624579	0.0025411	*CYM*
C22:6 n-3	BovineHD1000015166	10	50404371	A/C	0.1456	4.86E-08	0.005148315	0.00176711	–
C22:6 n-3	BovineHD1000029857	10	102483257	A/C	0.1181	3.48E-07	0.005129722	0.00198152	–
C22:6 n-3	BovineHD1400018498	14	66120964	A/G	0.1168	8.09E-08	0.005746942	0.0019963	–
C22:6 n-3	BovineHD2800003012	28	9852321	A/G	0.06044	3.59E-07	−0.006883573	0.00246427	–
Fatty acid groups									
SFA	BovineHD0600017761	6	64216527	A/G	0.0989	4.29E-07	2.276282318	0.454104	–
PUFA	BovineHD0500031844	5	110438965	A/G	0.06456	3.26E-07	0.629658268	0.126903	*BAIAP2L2, MAFF, TMEM184B*
MUFA	BovineHD0600017761	6	64216527	A/G	0.0989	8.95E-08	−2.641123459	0.505798	–
MUFA	BovineHD0600017773	6	64280345	C/A	0.1511	4.43E-07	−2.15015271	0.436519	–
n-6	BovineHD0500031844	5	110438965	A/G	0.06456	1.21E-07	0.5470253	0.148374	*BAIAP2L2, MAFF, TMEM184B*
PUFA/SFA	BovineHD0500031844	5	110438965	A/G	0.06456	5.56E-07	0.01371442	0.00280407	*BAIAP2L2, MAFF, TMEM184B*
PUFA/SFA	BovineHD0900022545	9	81183556	G/A	0.07005	5.48E-07	0.012964716	0.00264927	*MIR2284AA-4*
PUFA/SFA	BovineHD1500021109	15	73179761	A/C	0.4931	7.80E-08	−0.007313559	0.0013969	–

^a^minor allele/ major allele

^b^The SNP effect was the additive effect calculated by FarmCPU

^c^Positional/putative candidate gene

**Fig. 1:**
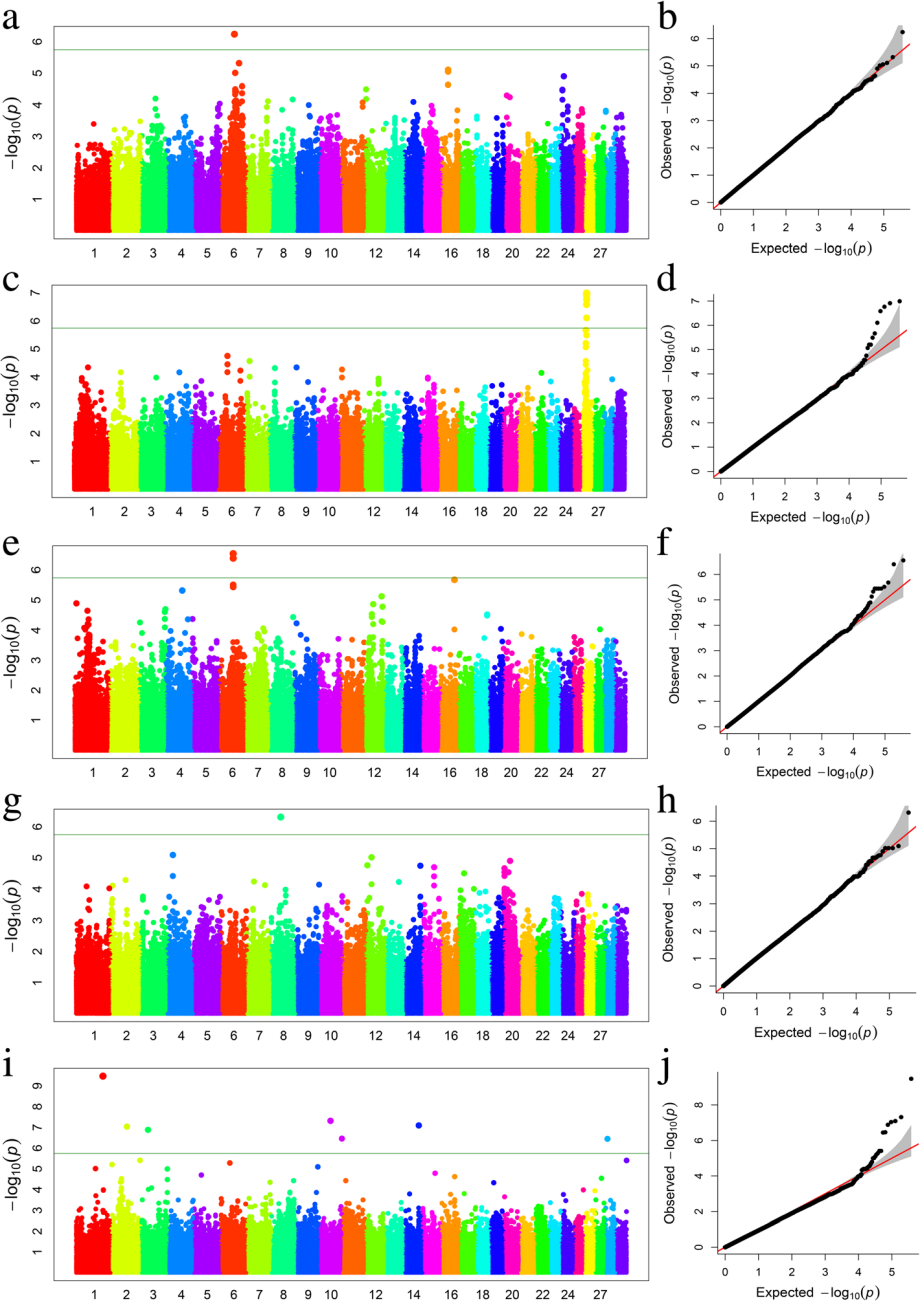
**a** Manhattan plot of association results for C22:0, where the *Y*-axis was defined as -log_10_(*P*) and the genomic position was represented along the *X*-axis. The green line indicated *P* = 1.36E-06. **b** Quantile-quantile plot of 503,579 SNPs in the genome-wide association study for C22:0. **c** Manhattan plot showing *P*-values of association for C14:1 *cis*-9. **d** Quantile-quantile plot for C14:1 *cis*-9. **e** Manhattan plot of association results for C18:1 *cis*-9. **f** Quantile-quantile plot for C18:1 *cis*-9. **g** Manhattan plot showing *P*-values of association for C20:1 *cis*-11. **h** Quantile-quantile plot of for C20:1 *cis*-11. **i** Manhattan plot showing *P*-values of association for C22:6 n-3. **j** Quantile-quantile plot for C22:6 n-3

**Fig. 2:**
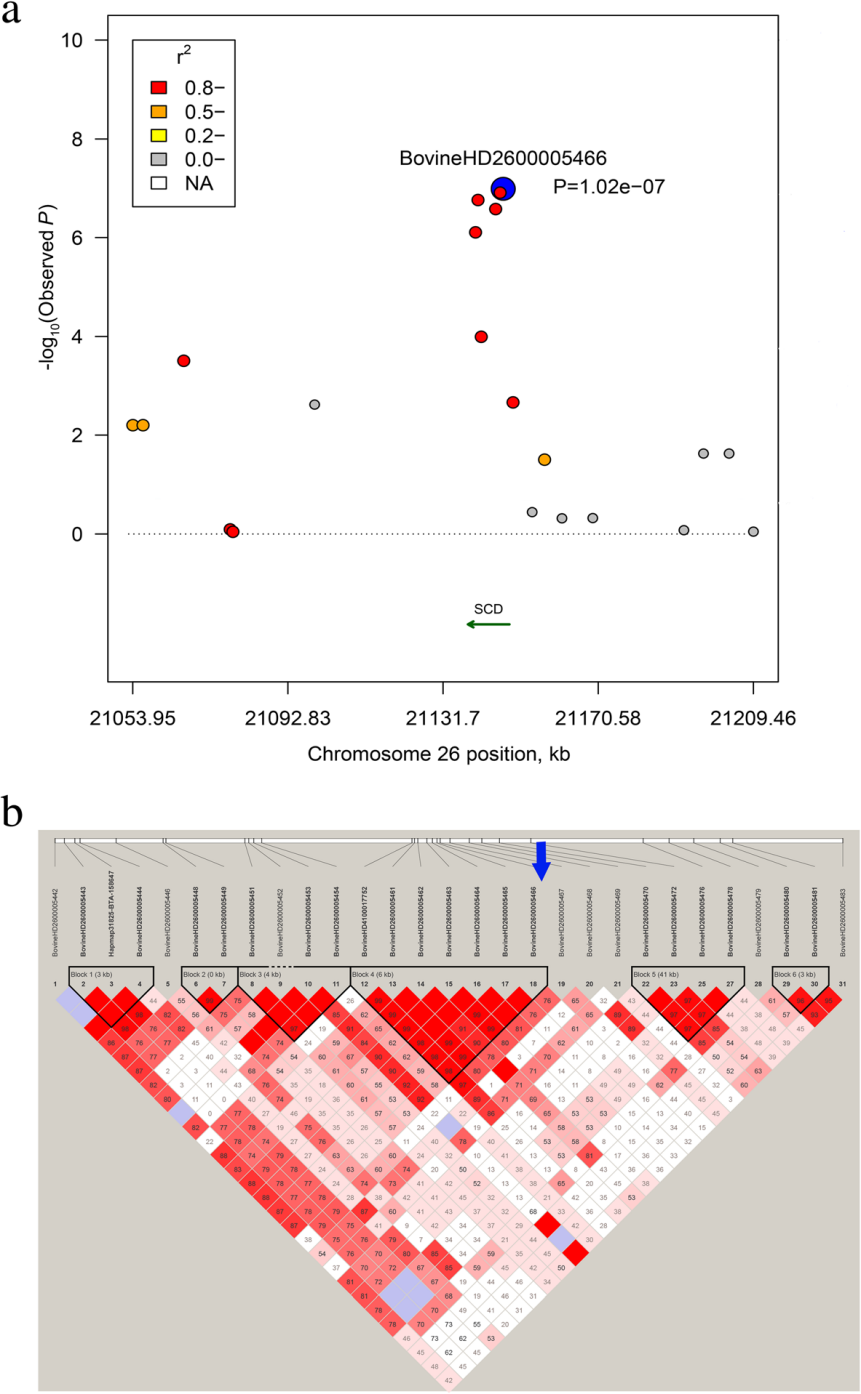
**a** Regional plots of candidate region at 21.05–21.21 Mb on BTA26 for C14:1 *cis*-9, the top SNP was highlighted by blue solid circles. Different levels of linkage disequilibrium (LD) between the most significant SNP and surrounding SNPs were indicated in different colors. **b** LD block for the region located at 21.05 Mb - 21.25 Mb on BTA26

Seven SNPs associated with C22:6 n-3 were detected that located at six chromosomes. Among them, three significant SNPs (*P*-value = 3.38E-10, 9.19E-08, and 1.31E-07) overlapped with phospholipid scramblase family member 5 (*PLSCR5*), CLIP-associating protein 1 (*CLASP1*), and Chymosin (*CYM*), respectively, while no genes were found for other four SNPs (Fig. 1i). Notably, we observed that four candidate SNPs at BTA26 within *SCD* for C14:1 *cis*-9 had significant *P* values (*P* = 1.02E-07) (Fig. 1c). Among them, three SNPs were imbedded within the intron of *SCD*, while one SNP located at its exon region. Our results revealed that the highly significant SNPs contribute ~ 7.37% of phenotypic variance for C14:1 *cis*-9, and some nearby SNPs display high LD around the *SCD* gene. In addition, we observed one block with 6 kb at this region (Fig. 2). Thus, these identified SNPs are possible candidate markers for further application of maker assisted selection. In addition, one SNP at 42.65 Mb in BTA8 (*P* = 4.89E-07) for C20:1 *cis*-11 overlapped with SWI/SNF Related, Matrix Associated, Actin Dependent Regulator of Chromatin, Subfamily A, Member 2 (SMARCA2) (Fig. 1g). We also found one SNP (at BTA6:63.66 Mb) associated with C22:0 (Fig. 1a) and two SNPs (at BTA6:64.22 Mb and BTA6:64.28 Mb) were associated with C18:1 *cis*-9, respectively (Fig. 1e). However, no putative candidate gene was identified near this SNP. Q-Q plots for five FAs were presented in Fig. 1b, d, f, h, and j.

**Fig. 3:**
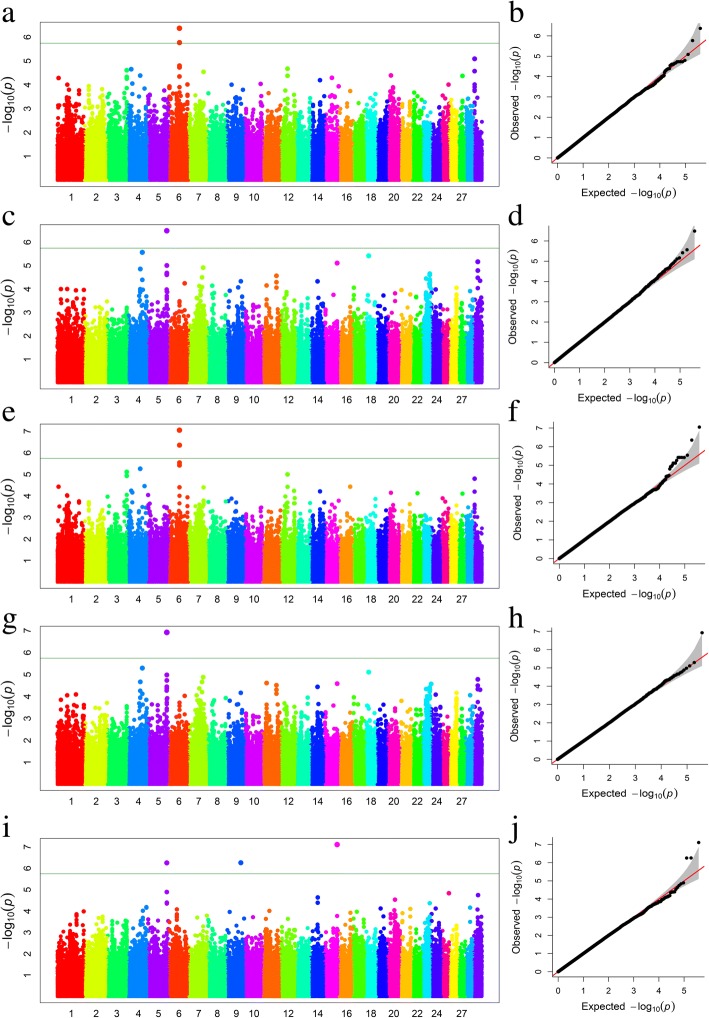
**a** Manhattan plot of association results for SFA, where the *Y*-axis was defined as -log_10_(*P*) and the genomic position was represented along the *X*-axis. The green line indicated *P* = 1.36E-06. **b** Quantile-quantile plot of 503,579 SNPs in the genome-wide association study for SFA. **c** Manhattan plot showing *P*-values of association for PUFA. **d** Quantile-quantile plot for PUFA. **e** Manhattan plot showing *P*-values of association for MUFA. **f** Quantile-quantile plot for MUFA. **g** Manhattan plot showing *P*-values of association for n-6. **h** Quantile-quantile plot for n-6. **i** Manhattan plot showing *P*-values of association for PUFA/SFA. **j** Quantile-quantile plot for PUFA/SFA

**Fig. 4:**
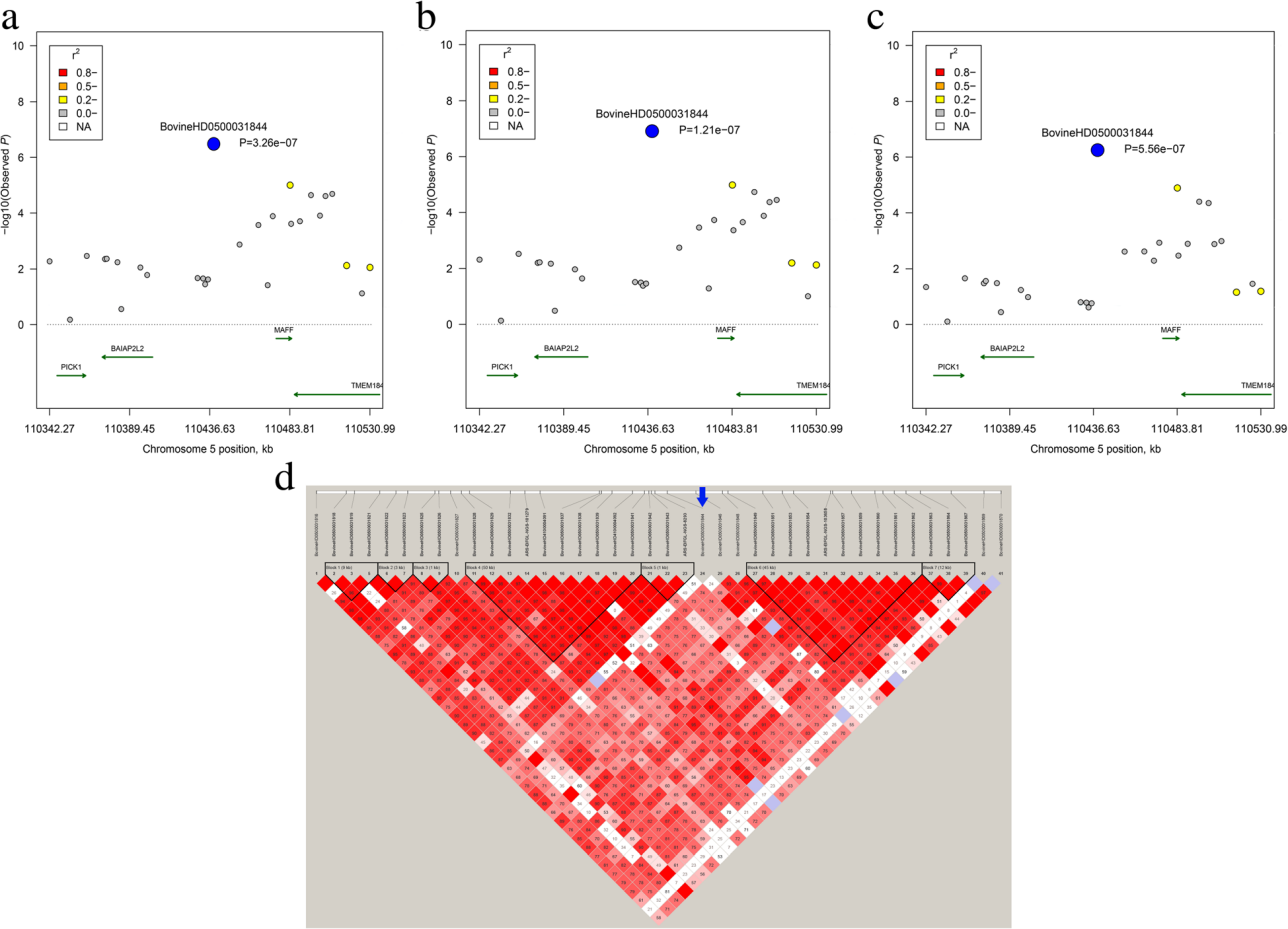
**a** Regional plot of candidate region at 110.34–110.53 Mb on BTA5 for PUFA. **b** Regional plot of candidate region at 110.34–110.53 Mb on BTA5 for n-6 group. **c** Regional plot of candidate region at 110.34–110.53 Mb on BTA5 for PUFA/SFA. **d** LD block for the region located at 110.34–110.53 Mb on BTA5

We also detected eight associated SNPs for five FA groups (Table 2 and Fig. 3). Of these SNPs, we observed three, two, one, one, and one candidate SNPs for PUFA/SFA, MUFA, PUFA, SFA, and n-6, respectively. QQ plots for five FA groups were presented in Fig. 3b, d, f, h, and j. Interestingly, we found one SNP (BovineHD0500031844) associated with pleiotropic effects for multiple FA groups (PUFA, n-6, and PUFA/SFA). Additionally, three candidate genes were identified at 100 kb widows around this SNP. Among them, one gene, *BAIAP2L2*, was located in the upstream region, while two genes, *MAFF* and *TMEM184B*, were located at the downstream region. Moreover, we found several large blocks around the significant SNPs, ranging from 110.3 to 110.5 Mb (Fig. 4d).

## Discussion

Fatty acids have generally been recognized as essential contributors to the tenderness and flavor of meat [46]. To our knowledge, this study is the first attempt to investigate the molecular mechanisms underpinning FAs using a high-density SNP array in Chinese Wagyu cattle. Our analyses showed that the estimated heritabilities varied among FAs, which is in agreement with previous publications [47–49]. This difference could be explained by the genetic architecture of the studied traits, and the effects of candidate SNPs may vary among diverse populations [25, 47]. In the present study, C14:1 *cis*-9 has the highest heritability (0.43) among MUFAs. Inoue et al. [50] and Ekine et al. [11] estimated that the heritability for C14:1 *cis*-9 were 0.86 and 0.51, which were also the highest among the MUFAs. These reports suggested that the amount of C14:1 *cis*-9 is likely to be influenced by genetic factors more than other MUFAs.

The heritability for C18:1 *cis*-9 was 0.25 in our analysis, whereas a relatively high heritability for C18:1 *cis*-9 (0.42 to 0.78) were reported in Japanese Black cattle [12, 32, 50]. Indeed, previous studies have reported that the SNPs within the *SCD* gene showing significant associations with C18:1 *cis*-13 of Canadian commercial steers as well as in Spanish breeds [51]. The heritability for eight FA groups were higher than those reported by previous studies [9, 10]. However, our results are similar to previous reports for the FA groups in Japanese Black cattle [12, 32, 50]. These results may suggest that the genetic structure of FAs in our population was more similar to Japanese Black cattle. The estimates of heritability for the FAs and FA groups are different across studies. This is particularly evident when the studied breeds are different, which may indicate differences in the genetic architecture of FAs in different populations. However, other factors, such as sample sizes and the statistical models, may also contribute to the difference of heritability estimates across studies [11]. We also observed high SE for some of FA except C16:0, C20:0, C18:2 n-6, C20:2 n-6 and C20:5 n-3, this is probably be explained by the small sample size. Also, population genetic structure and environmental conditions can affect the estimation of heritability.

For SFAs, strong positive genetic and phenotypic correlations between C22:0 and C20:0 were observed, which may indicate their similar origins of de novo synthesis from carbohydrates, amino acids, and volatile FA precursors [52]. The high positive phenotypic correlations between C22:0 and C20:0 also suggested that environmental conditions had similar effects on these FAs. Kim and Ntambi [53] proposed that C16:0 and stearic C18:0 fatty acids were the preferred substrates for *SCD* and can be converted to C16:1 *cis*-9 and C18:1 *cis*-9, respectively. C14:1 *cis*-9 is a major type of MUFA. We observed C14:1 *cis*-9 had a high positive genetic correlation with C16:1 *cis*-9, C18:1 *cis*-9, and C20:1 *cis*-9 (0.866, 0.789, 0.864), suggesting that the synthesis of MUFAs may be regulated by the same genes. C18:1 *cis*-9, as a type of FA proposed to be beneficial to human health, it had strong negative genetic correlations with the two most harmful saturated FA, C14:0 (−0.828) and C16:0 (−0.984). Genetic improvement of C18:1 *cis*-9 can possibly lead to a much healthier FA profile [5]. C16:1 *cis*-9, C18:1 *cis*-9, and C20:1 *cis*-9 also had strongly negative genetic correlations with C14:0, C16:0, C18:0, and C20:0. These results suggest that the synthesis of MUFAs may lead to a reduction of the concentration of SFAs.

In total, we identified 23 SNPs associated with nine candidate genes for FA composition in Chinese Wagyu cattle. Among them, we observed four significant SNPs for C14:1 *cis*-9 located at 21.14 Mb on BTA26, these newly identified SNPs were embedded with Stearoyl-CoA desaturase (*SCD*). Also, several LD blocks were observed within this gene (Fig. 2). *SCD* is the key enzyme involved in the endogenous synthesis of conjugated linoleic acid (*CLA*) and the conversion of saturated fatty acids into mono-unsaturated fatty acids (MUFA) in mammalian adipocytes [33]. Many SNPs near *SCD* have been reported, which are associated with C14:1 *cis*-9, C16:1 *cis*-9, C18:1 *cis*-9, and C18:2 n-6 in different cattle populations [6, 20, 23, 54]. For instance, two associated SNPs for C14:1 *cis*-9 were detected at 17.39 Mb and 17.58 Mb on BTA26 in Gifu cattle [6]. Three associated SNPs at 18.99~21.26 Mb on BTA26 for C14:1 *cis*-9, C16:1 *cis*-9, C18:1 *cis*-9, and C18:2 n-6 were detected in Angus and Hereford-Angus crossbred populations in Canada [23]. We suspected that different candidate SNPs for FA composition may be due to different genetic bases across populations. However, no significant SNPs around *SCD* was found that associated with C18:1 *cis*-9 in Chinese Wagyu cattle, which was consistent with previous findings in other populations [16, 20, 21, 51]. In addition, the direct effect of polymorphism within *SCD* on FA composition of milk has been extensively reported [6, 8, 18, 21–25]. Changes in the enzymatic activity as a result of *SCD* polymorphism and regulation have been recognized to cause diet-independent variations of CLA content in milk [55].

We also identified three genes, namely, CLIP-associating protein 1 (*CLASP1*), chymosin (*CYM*), and phospholipid scramblase family member 5 (*PLSCR5*) for C22:6 n-3. Among them, *CLASP1* located within a QTL region that was related to Warner-Bratzler shear force in Nelore beef cattle [56]. A previous study reported that the *CYM* is related to immune response and milk fat percentage [57]. In addition, we observed one SNP at 123 Mb in *PLSCR*, which was associated with C22:6 n-3. *PLSCR* has been previously reported to be related to reproductive traits in pigs [58, 59]. In our study, highly positive phenotypic and genetic correlations were observed between each pairwise comparison of PUFA, PUFA/SFA, and n-6. The phenotypic correlation coefficients between PUFA vs. PUFA/SFA, PUFA vs. n-6, and PUFA/SFA vs. n-6 were 0.825, 0.99, and 0.817, while the genetic correlation coefficients were 0.751, 0.948, and 0.632, respectively. These results suggest that FA groups with high positive correlations may be regulated by the same genes. Indeed, we identified three genes, including *BAIAP2L2*, *MAFF*, and *TMEM184B*, for multiple FA groups (PUFA, n-6, and PUFA/SFA), which may imply their pleiotropic effects for these traits in beef cattle. Among them, we found that *MAFF* has a high expression in fat tissues as reported by Fagerberg et al. [60]. Another gene, named *BAIAP2L2*, has been previously reported as related to the marbling score in Korean cattle, and the differentially expressed pattern of this gene is correlated with the expression of several miRNAs [61].

Previous studies suggested that Fatty acid synthase (*FASN*) and Elongation of very long chain fatty acids protein 5 (*ELOVL5*) are associated with fatty acid compositions including C14:0, C14:1 *cis*-9, C16:0, C16:1 *cis*-9, C18:0, and C18:1 *cis*-9 in cattle [8]. However, these two genes were not identified in the present study, thus we suspected that heterogeneous genetic architecture of fatty acids exists among different populations. We accurately detected one SNP at 120 Mb on BTA1 for C20:4 n-6, this SNP was embedded with *ELOVL7* (suggested *P-*value = 1.05E-05). In addition, several studies have found candidate SNPs within *ELOVL7* associated with FA composition in porcine muscle and abdominal fat tissues [62, 63]. In mammals, seven enzymes have been identified in the *ELOVL* family (*ELOVL1*–*7*). The *ELOVL* enzyme has a distinct distribution in different tissues, and different enzymes exhibit different preferences for the FA substrate. The *ELOVL5* and *ELOVL6* genes are involved in the production/synthesis of palmitic (C16:0), palmitoleic (C16:1 *cis*-9), stearic (C18:0), and oleic (C18:1 *cis*-9) fatty acids. Therefore, the role of *ELOVL5* and *ELOVL6* in the synthesis of FAs is of great importance for beef cattle breeding programs [24, 64, 65]. The investigation into the molecular mechanism of the *ELOVL* gene family can provide valuable insight into improving the composition of beneficial FA in cattle and expanding our knowledge of transcriptional regulation mechanisms in domestic animals [66].

## Conclusions

We identified several candidate SNPs and genes for individual FAs and FA groups in Chinese Wagyu cattle. Our findings highlight four novel SNPs in *SCD* with large effects for C14:1 *cis*-9 and one SNP with pleiotropic effects related to FA groups.

## Additional files

Additional file 1: **Table S1.** Estimates of phenotypic correlations (upper diagonals) and genetic correlation (lower diagonals) between 21 phenotypes in Chinese Wagyu beef cattle. (XLSX 199 kb)

## Abbreviations

BTA: *Bos taurus* autosome
FA: Fatty acids
GWAS: Genome-wide association study
HI: Health index
MUFA: Total monounsaturated fatty acids
n-3: Total omega-3 fatty acids
n-6: Total omega-6 fatty acids
n-6/n-3: Ratio between n-6 and n-3
PUFA: Total polyunsaturated fatty acid
QTL: Quantitative trait loci
SE: Standard error
SFA: Total saturated fatty acids
SNP: Single nucleotide polymorphism
